# Clinical Variation in the Treatment Practices for Medication Nonadherence, Drug–Drug Interactions, and Recognition of Disease Progression in Patients with Chronic Cardiometabolic Diseases: A Cross-Sectional Patient Simulation Study among Primary Care Physicians

**DOI:** 10.1155/2022/6450641

**Published:** 2022-07-30

**Authors:** Czarlota Valdenor, Divya Ganesan, David Paculdo, Joshua Schrecker, Rebecca Heltsley, Christopher Westerfield, John W. Peabody

**Affiliations:** ^1^QURE Healthcare, San Francisco, CA, USA; ^2^Aegis Sciences Corporation, Nashville, TN, USA; ^3^University of California, San Francisco, CA, USA; ^4^University of California, Los Angeles, CA, USA

## Abstract

**Background:**

Medication nonadherence in patients with chronic diseases is common, costly, and often underdiagnosed. In the United States, approximately 40–50% of patients with cardiometabolic conditions are not adherent to long-term medications. Drug–drug interactions (DDI) are also underrecognized and may lead to medication nonadherence in this patient population. Treatment complexity associated with cardiometabolic conditions contributes to increased risk for adverse drug events and DDIs.

**Methods:**

We recruited a nationally representative sample of 246 board-certified family and internal medicine physicians to evaluate how they assessed, identified, and treated medication nonadherence, DDIs, and worsening disease. Participating physicians were asked to care for three online simulated patients, each with at least one chronic cardiometabolic disease, including atrial fibrillation, heart failure, diabetes mellitus, or hypertension, and who were taking prescription medications for their disease. Physicians' scores were based on evidence-based care recommendation criteria, including overall care quality and treatment for medication nonadherence and DDIs.

**Results:**

Overall, quality-of-care scores across all cases ranged from 13% to 87% with an average of 50.8% ± 12.1%. The average overall diagnostic plus treatment score was 21.9% ± 13.6%. Participants identified nonadherence in just 3.6% of cases, DDIs in 8.9% of cases, and disease progression in 30.3% of cases.

**Conclusions:**

Based on these study results, primary care physicians were unable to adequately diagnose and treat patients with chronic cardiometabolic diseases who either suffered from medication nonadherence, DDIs, or progression of the disease. Improved standardization and technique in identifying these diagnoses is needed in primary care. *Trial Registration*. This trial is registered with clinicaltrials.gov, NCT05192590.

## 1. Introduction

Addressing chronic cardiometabolic disease is a major challenge for healthcare systems [1–3]. Despite wide-ranging efforts, patients do not always achieve optimal treatment targets [4]. A variety of factors could explain why, including medication nonadherence, drug–drug interaction (DDI) between medications or ingestants, or disease progression [2, 5].

Medication adherence is commonly defined as taking 80% or more of prescribed medication doses [6]. In the United States (US), approximately 40–50% of patients with chronic cardiometabolic conditions are not adherent to their long-term medications [6–8]. Nonadherence to prescribed treatment is thought to cause at least 100,000 preventable deaths and $100 billion in preventable medical costs per year [6–9]. One potential reason for this underdetection is most clinicians are unaware of the extent or consequences of nonadherence; many have never been formally trained for screening, diagnosing, or treating nonadherence [8]. In addition, complex factors surrounding medication nonadherence (patient beliefs, physician behaviors, healthcare system elements) make it a challenge for the clinician to identify and confirm the diagnosis [10]. The inability to correctly uncover nonadherence leads to unwarranted intensification of therapy, increasing the risk of adverse effects, raising health costs, and further lowering adherence due to higher pill burden and increased regimen complexity [10].

DDIs are also underrecognized and contribute to suboptimal pharmacologic therapy [11, 12]. DDIs occur when two or more drugs interact with each other, leading to additive or antagonistic pharmacological effects, influencing efficacy or adverse effects [12, 13]. Polypharmacy encountered in patients who often have multiple cardiometabolic conditions further contributes to increased DDI risk, adverse drug events, and poor outcomes [14].

In one study, more than 99% of primary care physicians (PCPs) said they ask their patients about drug reconciliation; however, objective measurement showed these physicians diagnosed DDIs in only 15.3% of patients [11]. Another study showed that in a selected patient population taking four or more drugs, 78.4% had a moderate to severe DDI [15]. Evidence shows that DDI testing in real-world patients significantly improves the management of polypharmacy, DDIs, and patient outcomes [16].

Disease progression is defined as the natural history of the disease, including any symptoms the patient is facing, biomarkers of the drugs used to combat such symptoms, and the specific action of the drug itself [17]. Even with appropriate treatment, chronic conditions may worsen [18], leading to higher morbidity and mortality. As such, treatment failure not attributable to nonadherence or DDI may be from ineffective therapeutics and/or worsening disease. To mitigate these effects, physicians will often adjust medication doses, prescribe more aggressive therapy, perform a more intensive diagnostic workup, increase risk factor monitoring, and/or adjust lifestyle habits to limit disease progression. Ruling out medication nonadherence and DDI is crucial for identifying disease progression and providing adequate and proper treatment to the patient.

Present methods for distinguishing between medication nonadherence, DDIs, and disease progression are limited [4, 19]. Currently, ways to identify nonadherence and DDI in patients include biomarker measurements, patient self-reports, pill counts, electronic monitors, and pharmacy alerts, with each approach having advantages and disadvantages [7]. None of these methods satisfactorily address the conundrum of nonadherence versus DDIs versus disease progression.

## 2. Methods

### 2.1. Overview

We conducted a prospective cohort, cross-sectional trial to observe how PCPs made clinical decisions in the assessment, diagnosis, and management of patients with chronic cardiometabolic diseases who were either nonadherent to their medications, had a DDI, or had suboptimal treatment response due to disease progression.

The study enrolled US-based practicing PCPs from October 2021 to December 2021. We evaluated medication nonadherence and DDI screening, workup, and care recommendations of board-certified family and internal medicine physicians as they cared for identical simulated patients through Clinical Performance and Value (CPV®) vignettes.

### 2.2. Ethics

This study was conducted following ethical standards, approved by the Advarra Institutional Review Board, Columbia, MD, USA, and listed in clinicaltrials.gov (NCT05192590). Informed consent was obtained from all participants before study enrollment.

### 2.3. Physician Selection

We recruited participants from a nationally representative list of over 30,000 PCPs. The recruitment lists were compiled from relevant contact resources, including medical association workforce databases and list serves, hospital organization physician rosters, and national medical conference attendees. Physicians were randomly invited to participate between October 2021 and December 2021. Physician participants were informed of the purpose of the study and, if they agreed to participate in the study, completed a brief screener before recruitment. Recruitment continued until a total of 246 participants were enrolled and completed their CPV® vignettes.

Physicians were deemed eligible for the study if they met the following criteria at the time of the study: they (1) are board-certified and currently practicing as family medicine or internal medicine physician; (2) have been practicing as a board-certified physician in internal or family medicine for between 2 and 35 years; (3) practice in a community or nonacademic setting; (4) care for more than 40 patients weekly; (5) commonly treat patients with cardiometabolic conditions such as atrial fibrillation, coronary artery disease, heart failure (HF), diabetes (DM), hypertension (HTN), and hyperlipidemia; (6) practice in the US; (7) speak English; (8) have Internet access; and (9) provide informed and voluntary consent into the study. The screener was distributed to the PCP list. Using the initial list and snowball sampling, we collected 622 potential participants who completed the participant screener. Of these, 261 met the inclusion criteria and consented to be enrolled in the study. Once consented and enrolled, physician and practice characteristics were gathered from a brief questionnaire. Of this 261 total, eight participants retracted consent and withdrew from the study, and seven participants did not complete all three of their cases. A total of 246 participants completed their three cases for a total of 738 patient cases.

### 2.4. Clinical Performance and Value (CPV®) Vignettes

We used CPVs to collect data on clinical practice variation. CPVs are validated online patient simulations widely used to measure clinical care [20, 21]. These vignettes include open-ended questions simulating typical clinical encounters and involve five domains: taking a patient medical history, performing a physical exam, ordering a diagnostic workup, making a diagnosis, and providing a treatment plan and follow-up. Evidence-based criteria outline each case. Two physicians, working independently, scored these cases using explicit, predetermined criteria. If scores were not unanimous between the two physicians, a third physician would independently serve as an adjudicator for the final score. Each domain, as well as the overall case, generated a quality-of-case percentage score between 0% and 100%. Higher percentage scores indicated greater adherence to the evidence-based criteria.

### 2.5. CPV Patient Cases

We created a total of nine CPV cases that simulated typical patients with chronic cardiometabolic conditions. The design followed a 3 × 3 matrix with three different patient disease types and three patient variants, based on the different risk factors that affect medication adherence and DDI. The disease types included patients with atrial fibrillation (AF), heart failure (HF), or diabetes/hypertension (DM/HTN). The variants reflected how these risk factors played in specific scenarios: case variant A featured patients who were nonadherent to their prescribed medication, case variant B patients had reduced therapeutic efficacy secondary to a DDI, and case variant C patients were adherent to medications, had no DDI, but had symptoms secondary to disease progression.

### 2.6. Analysis

The primary outcome of this study is to identify the baseline variation in clinical care among PCPs using standard-of-care tools. Specifically, we measured how physicians distinguished between nonadherence, reduced therapeutic efficacy secondary to DDI, and full compliance with suboptimal response to medication treatment. We also evaluated the demographics of providers and their clinical practice settings and analyzed how these variables impacted the workup, diagnosis, and management of medication nonadherence, DDI, and ineffective therapy care/progression of the disease. We modeled continuous outcome variables in a multiple linear regression model, controlling for potential confounders. We also modeled binary outcome variables using a logistic regression model. All analyses were conducted in Stata 14.2.

## 3. Results

### 3.1. Physician Characteristics

In total, 246 board-certified PCPs met the eligibility requirements and completed the physician questionnaire and the three CPV patient cases (Table 1). A slight majority of the sample specialized in internal medicine (54.1%) and most of the rest (45.1%) were boarded in family medicine with the balance being double-boarded in both. Males made up nearly three-quarters of participants (73.6%), and the mean (±SD) age was 56.4 ± 8.3 years old. Most of the physician participants worked in an urban or suburban setting (87.2%). By practice type, more than one in three (36.2%) worked in a primary care practice group, while 27.6% worked in a multispecialty practice, and 25.6% worked as solo practitioners. Over three-quarters (77.2%) of the study participants were employed by their practice, and nearly three in five (57.3%) received a quality bonus. The average payer mix for these PCPs was 47.2% commercial, 45.2% Medicare/Medicaid, 5.3% self-pay, and 2.3% other forms of payment. In addition, 99.0% of providers indicated they used some form of medication reconciliation in their everyday practice, with 88.2% saying they used pharmacy reconciliation, 79.3% using electronic medical record (EMR) alerts, and 69.5% using patient self-reports.

### 3.2. Variability of Physician Practice

**Figure 1:**
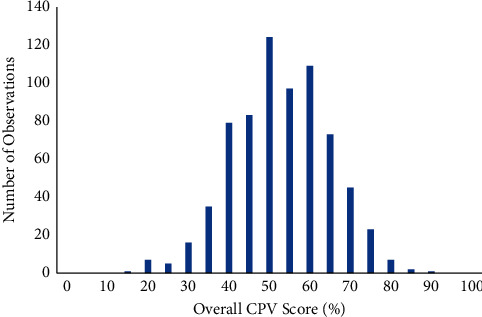
Overall CPV scores.

We found a wide variation in overall care in these patients with cardiometabolic diseases. The overall, quality-of-care scores across all cases ranged from 13% to 87% (Figure 1) with an average score of 50.5% ± 12.1% (Table 2). By disease area, we found that participants performed best in the HF cases (51.6% ± 13.0%), followed by DM/HTN (50.8% ± 12.0%) and AF (49.0% ± 11.7%). The difference in performance between HF and AF, while statistically significant (*p*=0.015) did not reach the 3–5% threshold of clinical significance [22].

Across the three case variants, we saw mean scores of 53.9% ± 12.7% for the variant A (nonadherence) cases, 48.4% ± 12.0% for the variant B (DDI) cases, and 49.2% ± 11.4% for the variant C (disease progression) cases. Overall scores were both statistically and clinically significantly higher in the variant A cases (*p* < 0.001).

### 3.3. Diagnostic and Treatment Accuracy

We calculated a diagnostic plus treatment (DxTx) score for the three case types. This score measures how well the providers did in making the correct diagnosis, identifying any comorbidities, and specifying the disease severity and in providing the correct treatment and follow-up. The overall DxTx score also ranged widely averaging 21.5% with a standard deviation of 13.5%. The DxTx score for DM/HTN was statistically significantly worse than the other two case types (19.7% ± 12.8% for DM/HTN, 22.4% ± 11.2% for AF, and 22.3% ± 16.0% for HF; *p*=0.025).

### 3.4. Distinguishing between Nonadherence, DDI, and Disease Progression

When documenting their patients' underlying cardiometabolic diseases, participants recorded the correct diagnosis in 87.4% of cases, ranging from 84.1% in the HF cases to 86.6% in the AF cases to 91.4% in the DM/HTN cases (*p*=0.045). However, when we looked at the different case variants, providers did not do nearly as well. Participants identified nonadherence (variant A) less than one time in 25 (3.3%), and they identified a DDI (variant B cases) about one time in 11 (8.2%). They were much better at distinguishing disease progression (variant C), correctly noting disease progression nearly one-third of the time (30.1%).

When we looked across all cases, physicians noted medication adherence as an important factor in their diagnostic consideration only 16.7% of the time, ranging from 20.4% in the nonadherence cases to 10.4% in the DDI cases, and 18.5% in the disease progression cases. Potential DDIs from polypharmacy were considered at an even lower rate (noted only 6.7% of the time), with little difference between the case variants.

### 3.5. Variant-Specific Analysis

We next investigated the most common incorrect diagnoses for each of the three variants. In the variant A cases, of the 96.7% of cases not diagnosed with NA, 78.8% in the AF case, and 87.2% in the DM/HTN case were incorrectly diagnosed with depression. In the HF nonadherence case, however, disease progression, and not depression, was incorrectly identified 62.3% of the time (Table 3). In the variant B cases, of the 91.9% of cases not diagnosed with DDI, 40.3% of the AF cases were diagnosed with hypertension, 35.1% in the HF case diagnosed with disease progression, and 46.3% in the DM/HTN case diagnosed with hypoglycemia. Finally, in the variant C cases, of the 69.9% of cases not diagnosed with disease progression, 91.7% in the AF case diagnosed mild cardioembolic stroke, and 69.0% of the DM/HTN case diagnosed with headaches related to stress/tension. In the HF disease progression case, 91.8% correctly diagnosed progression of HF.

A multivariate regression analysis on making the correct diagnosis showed no systematic physician or practice characteristics which proved significant. Within the individual case variants, we found some significant variations. Male physicians were significantly less likely to diagnose medication nonadherence (OR 0.1, 95% CI 0.0–0.7), and solo practitioners and providers in the west region were significantly more likely to identify disease progression (OR 2.1, 95% CI 1.1–4.0 and OR 2.7, 95% CI 1.3–5.4, respectively). There were no characteristics that made the identification of DDIs more likely.

The participants in this study performed poorly in providing advice to their patients on what was causing their symptoms, whether nonadherence, DDI, or disease progression. In the patients whose symptoms were caused by medication nonadherence, the physicians advised about the importance of adherence and interventions to increase adherence in only 5.3% of cases. In both the DDI and progression cases, the doctors spoke with their patients about the underlying reasons for their symptoms (either DDI or disease progression) in less than 1% of cases (0.4% for DDI and 0.6% for disease progression). Notably, the participating physicians performed somewhat better in ordering the primary treatment for their patients. The physicians ordered the continuation of the medication in nearly 8% of the variant A cases (7.8%) and stopped the interacting medication in about one-sixth of the variant B cases (16.3%) (Table 4). In the variant C cases, the physicians ordered the primary treatment in over one-fifth (20.7%) of the cases. For these cases, the primary treatment includes shifting medications due to medication resistance in the AF case, revascularization in the HF case, and intensive pharmacotherapy and workup for secondary HTN in the DM/HTN case. Treatment regimens were intensified via increased medication doses (13.1%) and/or adding or shifting to new medications (53.1%). In the same variant, medications were simply continued in 28.1% of cases and discontinued in 6.6%.

A multivariate regression analysis on primary treatment showed no systematic physician or practice characteristics which proved significant. Interestingly, making the correct cardiometabolic diagnosis was not significantly predictive of ordering the correct primary treatment. In fact, the only characteristic of significance was in the disease progression cases, where physicians in multispecialty practices were two-fifths as likely to make the primary treatment (OR 0.4, 95% CI 0.2–0.9).

## 4. Discussion

Effective pharmacologic therapy is crucial in improving patient outcomes and achieving cost savings. Medication nonadherence and DDIs affect a significant proportion of chronic cardiometabolic patients and can lead to adverse health events and costly hospitalizations [23]. Despite the health and economic consequences, there is a growing concern that diagnostic failures and persistent pharmacologic treatment errors are harming patients [24]. We conducted a study among a nationally representative sample of 246 PCPs caring for common types of patients with medication nonadherence, DDIs, or worsening disease.

We found that the recognition of medication nonadherence and DDIs is poor: participating physicians recognized medication nonadherence and DDIs only 3.3% and 8.2% of the time, respectively. Additionally, physicians only identified disease progression 30.1% of the time when it was the cause of inadequate treatment. These rates indicate that physicians struggle to identify and distinguish between medication nonadherence, DDI, and disease progression; that they may not be considering these different etiologies; and that they are unaware of the shockingly high prevalence of these causes for treatment inadequacy or failure.

Furthermore, our findings indicate that when nonadherence is missed, physicians are more likely to increase treatment. This trend was similarly observed in all case types. We also found that among the patients with nonadherence, physicians increased medication doses exposing the patients to more medication adverse effects and increasing treatment complexity, potentially leading to more nonadherence. This finding could indicate a lack of diagnosis in nonadherence leading to failure to provide the correct treatment for medication nonadherence.

In the simulated patients with DDIs, physicians mistakenly diagnosed nonadherence in 7.5% of all case types and diagnosed disease progression in 13.3%. When caring for patients with HF, physicians did worse in correctly diagnosing DDI, attributing the symptoms to nonadherence 12.4% of the time and to disease progression 33.7% of the time. While physicians did better at taking the correct follow-up action across case types (44.5% discontinued the interacting medication), there was still a significant proportion of those who did not correctly address the main etiology (27.4% opted to continue with the current regimen, 40.5% increased the medication doses, and 39.4% added or shifted to new medications). This inappropriate follow-on care could lead to persistent symptoms, more DDIs, increased rates of adverse effects, and inadequate treatment of the underlying condition.

Finally, in patients with disease progression, physicians were unable to properly distinguish between disease progression, medication nonadherence, and DDI in patients with diabetes/hypertension, incorrectly diagnosing nonadherence and DDI 3.6% and 2.4% of the time, respectively. This inability leads to delays in the optimization of treatment regimens and worse patient outcomes.

From our data, HF patients were particularly problematic as physicians struggled to distinguish between these three explanations of therapeutic shortfalls. Although patients with atrial fibrillation or diabetes/hypertension still faced issues in receiving the correct diagnosis and follow-on care, HF patients were significantly more likely to be incorrectly diagnosed and treated. Part of the reason is due to clinicians not considering DDIs, polypharmacy, or medication adherence as an issue, identifying them only 6.7% of the time. Moreover, the data indicate saturation in incorrectly diagnosing patients with disease progression, perhaps because that may be the easiest case for physicians to check. To limit incorrect diagnoses of disease progression, a more reliable method to distinguish these three conditions is needed.

The participating physicians did well in identifying the patients' baseline chronic diseases. When documenting their patients' chronic cardiometabolic diseases, participants recorded the correct diagnosis in 86.9% of cases, ranging from 83.1% in the HF cases to 86.1% in the AF cases, and 91.5% in the DM/HTN cases (*p*=0.028). They had a good rate of identifying the patients' comorbid conditions, but not the acute problem (nonadherence, DDI, or disease progression). We interpret this finding as a gap between diagnosis and identifying the risks associated with nonadherence and possible DDIs and, thus, appropriate treatment advice. Notwithstanding, even when they were able to identify nonadherence, they did not always take steps to correct the problem. In contrast, physicians were more adept at discontinuing interacting medications (done in 44.5%) even when they have not explicitly diagnosed the DDI (correct diagnosis 8.2%), which may reflect some familiarity with the usually available practice tools that help identify potential DDIs. However, this number is still low and warrants further improvement.

Diagnosing medication nonadherence and DDIs has proven to be tricky since it relies too heavily on subjective information (patient's memory of taking their medications, them detailing potential side effects after taking the medication) [8, 10, 25]. This information is compounded by the lack of standardized practice and training in physicians' abilities to diagnose and treat these diseases [10, 25]. Notwithstanding these worrisome findings, most study participants reported using tools for medication reconciliation in their everyday practice, with 89.2% saying they used pharmacy reconciliation, 79.3% using EMR alerts, and 70.3% using patient self-reports. Ultimately, current methods of diagnosing adherence and DDI do not allow physicians to reliably make a definitive diagnosis distinguishing nonadherence from DDIs from disease progression, and it is not surprising that we did not see appropriate prescribing changes. There is a need for a more accurate, valid, and standardized method to diagnose medication nonadherence and DDI, as well as provide the physicians with reliable information to optimize treatment regimens appropriately for better outcomes.

Some potential limitations of this study include that our sample was 73.1% male physicians compared to the current gender distribution of primary care physicians in the US of 54% males [26]. Additionally, we did not look for every type of DDI; we investigated only the most common ones. This study only investigated primary care physicians, whereas future studies could explore how specialists handle these types of cases. We also studied specifically symptomatic patients, but medication nonadherence and DDI may be present in asymptomatic patients as well.

## 5. Conclusion

When PCPs were exposed to simulated patients with chronic cardiometabolic diseases, polypharmacy, and the presence of medication nonadherence, DDI, or disease progression, we found massive variation in diagnosis and treatment. Physicians failed to identify and appropriately diagnose their patients with medication nonadherence or DDI greater than 90% of the time, and only accurately identified disease progression 30.1% of the time, indicating the need for better identification of these conditions. As such, we should use the findings from this study to reveal physicians' inability to distinguish between medication nonadherence, DDI, and disease progression to study the best ways in which we can change and improve patient care. The first step, described in this paper, is to identify the problem. We conclude that medication reconciliation, physician education, and other current approaches are not adequate to identify and correct these shortcomings. We hypothesize that routinely checking for nonadherence and DDI is worth evaluating and may have enormous clinical and economic benefits.

## Figures and Tables

**Table 1:** Physician baseline characteristics.

	Overall
*N*	246
Male	73.6%
Age	56.4 ± 8.3
Internal medicine	54.1%
Number of years in clinical practice	
2–10	3.2%
11–20	28.9%
21–35	67.9%
Number of active patients in the panel	
500–1000	14.6%
1001–2000	38.6%
>2000	46.8%
Practice type	
Hospital-based	10.6%
Private, multispecialty	27.6%
Private, single specialty	36.2%
Private, solo	25.6%
Region	
Northeast	28.2%
South	32.0%
Midwest	22.0%
West	17.8%
Setting	
Urban	30.3%
Suburban	56.9%
Rural	12.9%
Employed by practice	77.2%
Payer, %	
Medicare	35.8%
Medicaid	9.4%
Commercial	47.2%
Self	5.3%
Other	2.3%
Participant in CMS quality program	
Yes	50.6%
No	35.7%
Do not know	14.1%
Receive quality bonus	57.3%

**Table 2:** CPV scores and diagnoses.

	Total CPV score (%)	Diagnosis + treatment (DxTx) CPV score (%)	Primary diagnosis (%)	Case variant diagnosis (%)
Overall	50.5 ± 12.1	50.5 ± 12.1	87.4	—
By case area				
Atrial fibrillation	49.0 ± 11.7	22.4 ± 11.2	86.6	86.6
Heart failure	51.6 ± 13.0	22.3 ± 16.0	84.1	84.1
Diabetes/hypertension	50.8 ± 12.0	19.7 ± 12.8	91.4	91.4
By case variant				
Nonadherence	53.9 ± 12.7	23.9 ± 13.6	—	3.3
Drug–drug interaction	48.4 ± 12.0	17.3 ± 14.1	—	8.2
Disease progression	49.2 ± 11.4	23.2 ± 11.9	—	30.1

**Table 3:** Incorrect diagnosis of nonadherence versus DDI versus disease progression by case variant.

Case	Incorrectly diagnosed as nonadherence (%)	Incorrectly diagnosed as DDI (%)	Incorrectly diagnosed as disease progression (%)
Variant A—nonadherence (overall)	—	0	19.1
1A (atrial fibrillation)	—	0	0
2A (heart failure)	—	0	62.3
3A (diabetes/hypertension)	—	0	0
Variant B—DDI (overall)	7.5	—	13.3
1B (atrial fibrillation)	1.3	—	0
2B (heart failure)	12.4	—	33.7
3B (diabetes/hypertension)	8.1	—	0
Variant C—disease progression (overall)	1.3	1.8	—
1C (atrial fibrillation)	0	0	—
2C (heart failure)	0	3.0	—
3C (diabetes/hypertension)	3.6	2.4	—

**Table 4:** Medication changes by case variant.

Case	Continue (%)	Decrease/stop	Increase (%)	Add/shift (%)
Variant A—nonadherence	**17.8**	n/a	24.4	17.8
Variant B—DDI	27.4	**16.3%**	40.5	39.4
Variant C—disease progression	28.1	6.6%	**13.1**	**53.1**

Physicians could perform more than one action. Bolded items are correct actions.

## Data Availability

The data used to support this study are available from the corresponding author upon request. Data were collected from CPVs®, QURE Healthcare's proprietary simulated case tool.
